# Does humeral head size predict the lateralization required to preserve near-anatomic posterosuperior rotator cuff length in reverse shoulder arthroplasty?

**DOI:** 10.1016/j.jseint.2026.101673

**Published:** 2026-02-26

**Authors:** Erica Lante, William G. Blakeney, Stefan Bauer

**Affiliations:** a Centre Hospitalier Universitaire Vaudois (CHUV), Lausanne, Switzerland; b Department of Orthopaedics and Trauma, Royal Perth Hospital, Perth, Australia; c Medical School, Division of Surgery, University of Western Australia, Perth, Australia; d Centre de l’Épaule, du Coude et du Membre Supérieur, Service d'Orthopédie et Traumatologie, EHC, Morges, Switzerland

**Keywords:** Reverse shoulder arthroplasty, Humeral head size, Lateralization, Posterosuperior rotator cuff, External rotation, Computational modeling, Implant design, Three-dimensional modeling

## Abstract

**Background:**

Reverse total shoulder arthroplasty (rTSA) lacks an anatomy-based framework for individualized implant planning. We hypothesized that native humeral head size (HHS) could serve as a patient-specific anatomical reference to guide lateralization in rTSA. The purpose of this study was to determine whether HHS predicts the amount of baseplate lateralization required to preserve near-anatomic posterosuperior rotator cuff muscle length, and whether muscle lengths can be reliably estimated in deformed joints using a statistical shape model (SSM).

**Methods:**

In this computational study, 83 pre-operative computed tomography scans were analyzed using a validated SSM to estimate rotator cuff muscle–tendon lengths. Five rTSA implantation strategies were modeled while controlling implant geometry, ranging from a medialized Grammont-type configuration to lateralized Frankle-type and hybrid strategies. Best-fit humeral head size was correlated with patient-specific lateralization quantified by overlap-based graft thickness and with posterosuperior cuff muscle length.

**Results:**

Best-fit humeral head size demonstrated an excellent correlation with graft thickness required for near-anatomic overlap (r = 0.93) and strong correlations with posterosuperior cuff muscle length, particularly the infraspinatus (r = 0.88), as well as the subscapularis (r = 0.76) and teres minor (r = 0.62). Among implantation strategies, the Frankle-type configuration most closely reproduced near-anatomic graft thickness and posterosuperior cuff muscle length. Implantation strategy produced large to very large differences in baseline muscle–tendon length across configurations (Kendall W ≈ 0.9-0.97). The SSM reliably reconstructed muscle–tendon geometry across a broad spectrum of degenerative pathology.

**Conclusion:**

HHS predicts the lateralization required to preserve near-anatomic posterosuperior cuff muscle length in rTSA. A Frankle-type lateralization strategy most closely reproduced the overlap-based anatomic reference, while alternative strategies produced systematic deviations in muscle length. These findings support HHS as an anatomy-based, patient-specific guide for lateralization planning. This framework is geometric and conceptual in nature and requires future clinical validation.

Reverse total shoulder arthroplasty (rTSA) has become the most commonly performed type of shoulder arthroplasty worldwide, now comprising nearly 90% of all procedures.^17^ Despite its prevalence, surgical planning for rTSA has yet to adopt a fully validated, anatomy-based framework for individualized implant configuration, even as computed tomography (CT)–based, patient-specific anatomical modeling and virtual implant positioning are increasingly used. These tools primarily support visualization and definition of implant orientation and alignment rather than providing a validated framework for individualized implant configuration. Accordingly, this gap in modern practice reflects the absence of a validated, anatomy-based framework for individualized implant configuration.^9^^,^^27^^,^^31^

Patients undergoing rTSA present with wide variability in humeral head size, glenohumeral alignment, and rotator cuff integrity. Nevertheless, a largely standardized approach to implant selection is commonly applied across a wide spectrum of patient morphologies and pathologies. This lack of anatomical scaling introduces biomechanical risk at both extremes of the size distribution. In larger cuff deficient shoulders, insufficient lateralization may lead to instability, and diminished muscle efficiency.^16^ In contrast, smaller shoulders are prone to overstuffing^2^^,^^5^^,^^6^ These opposing risks underscore the need for a scalable, anatomy-driven approach to rTSA configuration.

The original Grammont-type rTSA improved deltoid leverage by medializing the center of rotation^10^^,^^13^ but at the cost of rotator cuff shortening, altered muscle length relationships, and reduced rotation. To address these limitations, Frankle et al developed a more anatomic, lateralized rTSA concept aimed at restoring soft-tissue balance through geometry that more closely approximates native joint anatomy.^12^^,^^28^ This evolution aligns with Neer's foundational principle that restoration of native anatomy is central to optimizing shoulder biomechanics.^22^

Within this context, humeral head size (HHS) has emerged as a reproducible three-dimensional (3D) anatomical parameter that scales predictably with the overall glenohumeral size and morphology.^4^ HHS may provide a practical anatomical reference for the amount of lateralization required to preserve premorbid muscle length relationships. However, contemporary implant systems typically offer baseplate lateralization in fixed increments (eg, 0, 3, or 6 mm), without a standardized method for scaling these options to individual patient anatomy. Prior studies have demonstrated nonuniform scaling of glenohumeral dimensions across patients, resulting in distinct size groups and heterogeneous morphology.^11^ Such variability also affects muscle fiber length and moment arms, further highlighting the limitations of generalized lateralization strategies.^16^

Accurate assessment of muscle length in rTSA candidates is challenging, particularly in the presence of deformity, decentering, or chronic rotator cuff pathology. Advances in statistical shape models (SSMs), however, now allow reliable reconstruction of 3D musculoskeletal geometry and estimation of muscle–tendon paths even in altered anatomy.^24^ Advanced planning software enables patient-specific analysis of muscle–tendon geometry across standardized arm positions, providing a more anatomically meaningful assessment than static baseline configurations alone.^26^

To our knowledge, no prior study has systematically evaluated whether native HHS can predict the amount of lateralization required to preserve near-anatomic posterosuperior rotator cuff muscle length across different rTSA implantation strategies while controlling implant geometry. Accordingly, we analyzed 83 CT-derived shoulder models to address the following question: does HHS predict the baseplate lateralization required to preserve near-anatomic posterosuperior cuff muscle length in rTSA? In addition, we evaluated whether muscle lengths can be reliably estimated in deformed joints using validated SSM.

## Materials and methods

### Patient selection and imaging

This translational basic science computer modeling study used deidentified CT scans from 83 patients (Table I), approved by the ethics committee (CER-VD, Project-ID 2023-01051). CT data were analyzed using SurgiCase software (version 4.1; Materialise, Leuven, Belgium). Bony segmentation was performed automatically and refined manually using validated thresholds. Pre-operative glenohumeral parameters were calculated using a 3D SSM.^1^^,^^19^^,^^20^^,^^25^

**Table I:** Patient demographics and anatomical characteristics by diagnosis group.

Characteristics/diagnosis groups	All (n = 83)	OA (n = 23)	MRCT (n = 31)	CTA (n = 29)
Age (yr)	74 (50-90)	73 (59-85)	73 (50-83)	75 (54-90)
Gender (F/M)	44/39 (53%/47%)	13/10 (57%/43%)	18/13 (58%/42%)	13/16 (45%/55%)
Height (cm)	166 (135-189)	170 (156-185)	164 (145-185)	166 (135-189)
Weight (kg)	78 (39-128)	87 (60-127)	74 (47-120)	75 (39-128)
BMI (kg/m^2^)	28 (17-47)	30 (22-43)	27 (18-47)	27 (17-43)
Glenoid inclination (°)	7 (−15 to 28)	5 (−15 to 14)	9 (1-17)	7 (−3 to 28)
Glenoid retroversion (°)	9 (0-33)	12 (0-33)	6 (0-15)	10 (1-27)
Best-fit HHS (mm)	49 (40-56)	50 (46-56)	48 (42-53)	49 (40-55)
Anatomic osteotomy HHS (mm)	47 (38-54)	48 (44-54)	46 (40-51)	47 (34-53)
Humeral subluxation (%)	61 (24-88)	64 (24-88)	57 (41-78)	62 (40-85)
Vault loss (%)	8 (0-54)	13 (0-54)	2 (0-14)	10 (5-29)
Erosion depth (mm)	3 (0-11)	4 (0-11)	1 (0-3)	4 (2-10)

*BMI*, body mass index; *F*, female; *M*, male; *OA*, osteoarthritis; *MRCT*, massive rotator cuff tear; *CTA*, cuff tear arthropathy; *HHS*, humeral head size.

### Software-based segmentation and anatomical reference definitions

All bony segmentation and pre-operative anatomical measurements were performed using SurgiCase software (version 4.1; Materialise, Leuven, Belgium), following previously published and validated workflows. Cortical and cancellous bone were segmented automatically using standardized Hounsfield unit thresholds and refined manually when required, as previously described.^1^^,^^25^ Glenoid version and inclination were calculated using a 3D scapular coordinate system based on the Friedman line and scapular plane, in accordance with established methods reported in the literature.^19^^,^^25^ Glenosphere orientation was maintained neutral relative to the baseplate. Humeral version was derived from patient-specific humeral morphology relative to the humeral shaft axis using the software's standard geometric definitions.^1^ All angular measurements were reported in degrees, with neutral (0°) defined as alignment with the corresponding anatomical reference axis or plane of the defined coordinate system. These methods were applied consistently across all patients and implantation strategies in the present study.

### Modeling of implant configurations

Five distinct rTSA implantation strategies were modeled using a modular meta-epiphyseal implant system (Delta Xtend, DePuy Synthes, Warsaw, IN, USA) positioned stemless and inlay (strategies 1-5, Table II). The classic Grammont configuration was modeled using a 42-mm eccentric glenosphere with a high-mobility liner without baseplate lateralization. All other configurations were modeled using a constant near-anatomically lateralized 27-mm baseplate and 38-mm glenosphere with a standard liner. This approach was chosen intentionally to avoid additional geometric confounders and to isolate the geometric effect of the neck-shaft angle (NSA) and baseplate position on muscle–tendon length across lateralized implantation strategies.^3^ The NSA and the baseplate position were varied, with the baseplate modeled either centered on the glenoid surface or flush with the inferior glenoid rim. The standardized implant geometry enabled comparison of implantation strategies. Different commercially available implant designs were deliberately not included.

**Table II:** Overview of implant configurations modeled, including NSA, constraint, and lateralization type.

Model number, name, and description	Original meta-epihyseal NSA	Meta-epiphyseal tilt	Effective NSA	Liner type and LSR, DRR	BP position	BP size, lateralization, glenosphere size, and inferior overhang	Lateralization strategy with adjustment
1 classic Frankle-type, 135°, lateralized, centered BP (high), Near anatomic	155°	20°	135°	+3 mm standard, 147%, 0.44	High (centered)	27 mm BP lateralized (anatomical overlap), 38 mm, overhang: 5.5 mm	Lateral overlap if centered; overlap + (graft thickness − reaming depth)/2 if decentered
2 hybrid Frankle-type, 135° lateralized, distalized BP (flush)	155°	20°	135°	+3 mm standard, 147%, 0.44	Flush (inferior)	27 mm BP lateralized (anatomical overlap), 38 mm, overhang: 5.5 mm	Same as model 1
3 Hybrid-type, 145° lateralized, distalized BP (flush)	155°	10°	145°	+3 mm standard, 147%, 0.44	Flush (inferior)	27 mm BP lateralized (anatomical overlap), 38 mm, Overhang: 5.5 mm	Same as model 1
4 Lateralized Grammont 155° lateralized, distalized BP (flush)	155°	0°	155°	+3 mm standard, 147%, 0.44	Flush (inferior)	27 mm BP lateralized (anatomical overlap), 38 mm, overhang: 5.5 mm	Same as model 1
5 Classic Grammont-type, 155° medialized, distalized BP (flush)	155°	0°	155°	+3 mm high mobility, <147%, <0.45	Flush (inferior)	27 mm BP medialized, 42 mm + 2 mm eccentric, overhang: 9.5 mm	No lateralization; COR medialized

*BP*, baseplate; *NSA*, neck-shaft angle; *LSR*, liner stability ratio; *DRR*, depth-radius ratio; *COR,* centre of rotation.

The term Frankle-type refers to a centered baseplate 135° implantation strategy rather than to a proprietary device. The term hybrid refers to a distalized, lateralized baseplate implantation combined with a 135° or 145° NSA. For the humeral component, medial implant tilt was adjusted from 0° to 20° to model NSAs of 155°, 145°, and 135°. A consistent inlay humeral implant was used to isolate strategy-related geometric effects. Humeral osteotomy height was standardized to 19 ± 2 mm. Retroversion was automatically set at −20°, based on humeral morphology.^19^ Transepicondylar axis alignment was not used, as the elbow was not included in the scans. Implant positioning was confirmed by consensus between 2 fellowship-trained shoulder surgeons.

### Baseplate position and lateralization

**Figure 1:**
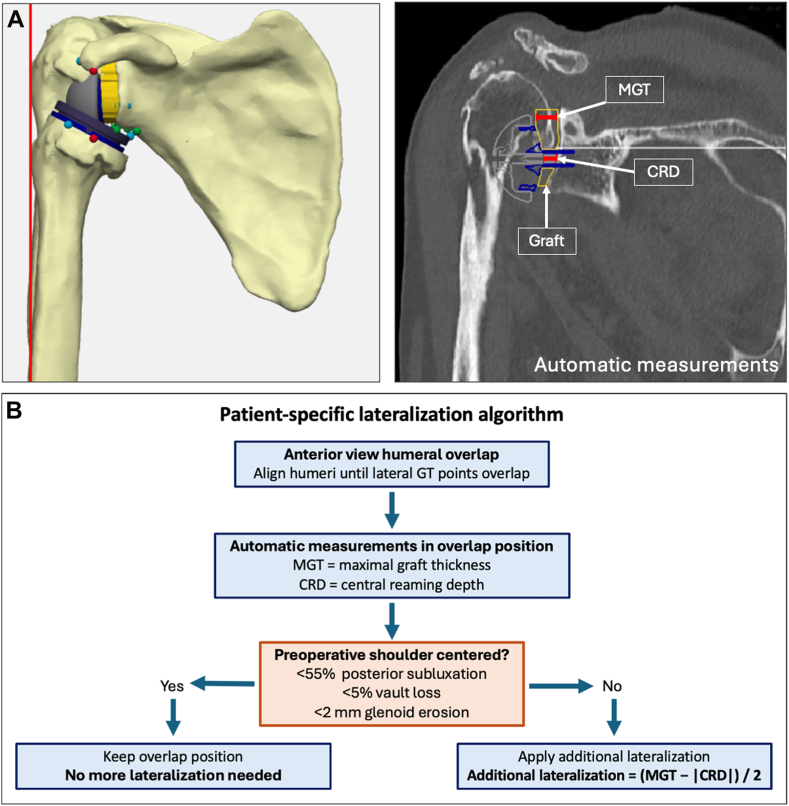
Planning strategy for additional lateralization. (**A**) Overlap-based lateralization concept, illustrated using a lateralized Grammont design with a 155° neck-shaft angle for visualization purposes. (**B**) Flowchart illustrating the planning process, applied uniformly across all implantation strategies. *MGT,* maximum graft thickness; *CRD,* central reaming depth; *GT,* greater tuberosity.

Baseplate positioning was standardized according to implant design. For the Frankle-type model, the baseplate was centered along the superior–inferior axis of the glenoid, positioned equidistantly between the highest and lowest points of the glenoid surface. For designs with an inferior flush baseplate, the component was positioned flush with the inferior glenoid rim. Anteroposterior positioning was defined, with the baseplate positioned equidistantly between the most anterior and most posterior points of the glenoid surface. All positioning parameters and measurements were achieved by consensus between 2 experienced shoulder surgeons. For consistency, all baseplates were implanted with 0° of inclination and 5° of retroversion relative to the Friedman-based scapular coordinate system. All configurations except the Grammont design used a lateral-glenoid/medial-humerus strategy to standardize glenoid lateralization and isolate the geometric influence of baseplate position and NSA on muscle–tendon length. Medial-glenoid/lateral-humerus strategies were not modeled, as their potential lever-arm benefits^15^ require dynamic, force-based modeling beyond the scope of the present study. Implant configuration details are summarized in Table II. To quantify patient-specific lateralization, the pre-operative and rTSA-configured humeri were superposed until the most lateral point of the greater tuberosity coincided in the anterior view. The amount of graft required to achieve this overlap, defined as near-anatomic lateralization, was measured as a negative reaming distance. Maximal graft thickness was recorded as the greatest structural graft thickness required to restore overlap, and central reaming depth as the negative value of central graft thickness (Fig. 1*A*).

Shoulders were classified as centered when software measurements showed humeral subluxation between 45% and 55%, <5% vault loss, and <2 mm of glenoid erosion. Cuff tear arthopathy shoulders with Hamada grade ≥ 2 and osteoarthritic shoulders with >2 mm of erosion were classified as decentered. For centered shoulders, anatomic humeral overlap was applied directly. For decentered shoulders, additional lateralization was applied to compensate for pathological medialization or subluxation, calculated as: additional lateralization = (maximal graft thickness − central reaming depth)/2 (Fig. 1*B*).

### Liner stability ratio and depth-radius ratio

Standard 38-mm liners (liner stability ratio^7^^,^^21^: 147%; DRR^18^: 0.44) were used for all lateralized models. The Grammont design was modeled with a high-mobility liner (LSR <147%, DRR <0.45). Liner thickness was fixed at 3 mm.

### SSM of muscle length

**Figure 2:**
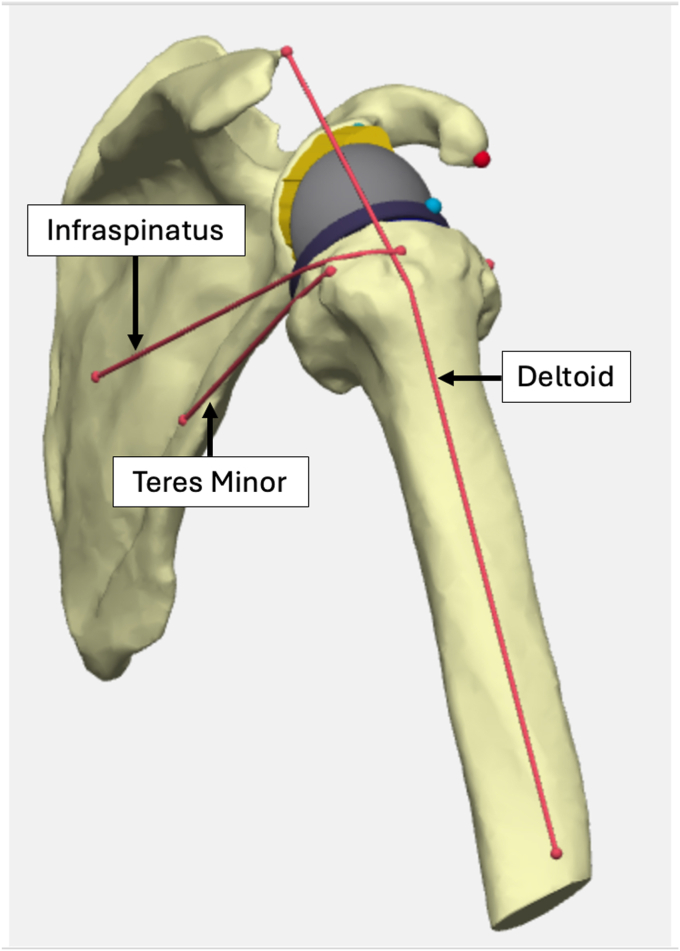
Stylized three-dimensional muscle length modeling, illustrated from a posterolateral view, showing the wrapping of the deltoid, infraspinatus, and teres minor on the same lateralized Grammont configuration for illustrative purposes.

A validated SSM was fitted to each patient's anatomy using automated registration and nonrigid deformation algorithms.^24^ Muscle origin and insertion points were transferred from the atlas model to patient-specific geometry using anatomical landmarks. Rotator cuff paths were reconstructed by connecting these points and wrapping them anatomically in 3 dimensions around the joint (Fig. 2). The SSM has previously demonstrated high fidelity, including in shoulders with osteoarthritis and posterosuperior subluxation.^24^ Reliable estimation of muscle–tendon length in deformed joints was a prerequisite for evaluating anatomy-based lateralization strategies.

### Arm position

Muscle–tendon lengths were assessed by comparing pre-operative and post-operative values, expressed as percentage change, using the original CT acquisition pose. Absolute muscle lengths (mm) were then measured in a standardized neutral arm position, defined as 0° forward flexion, 0° rotation, and 20° abduction. This neutral position was chosen to ensure a uniform arm configuration across patients and to allow direct comparison of muscle lengths. Functional arm positions and scapulothoracic motion were not analyzed, as the focus of the study was on geometric muscle–length relationships at rest.

### Outcome parameters

Primary outcomes included absolute and relative posterosuperior rotator cuff muscle–tendon length (mm, %) and patient-specific lateralization (mm) derived from overlap-based graft thickness. Best-fit humeral head size (BFHHS; mm) was computed using SurgiCase software and served as the anatomical reference for lateralization scaling. Anatomic osteotomy HHS was calculated using Glenosys (v10.6.4; Imascap, Brest, France) and correlated with BFHHS. BFHHS represents a geometric best-fit construct derived from native humeral morphology, whereas anatomical head cut size reflects a surgical measurement based on the planned osteotomy plane. The 2 parameters were intentionally obtained using different software platforms, as they describe distinct but complementary anatomical concepts. Secondary parameters included vault loss (%), erosion depth (mm), humeral subluxation (%), glenoid version and inclination (°), and baseplate height (centered vs. flush).

### Statistical analysis

All analyses were performed in Python (v3.11) using the statsmodels, scipy, and matplotlib libraries. A two-tailed alpha of 0.05 defined significance. Friedman chi-square statistics (χ^2^) were reported to analyze differences in muscle–tendon lengths across implant configurations. Effect sizes were reported as Kendall W (≥ 0.1: small, ≥ 0.3: moderate, ≥ 0.5: large). Pairwise comparisons between implant configurations were performed using the Wilcoxon signed-rank test for non-normally distributed, paired data.

Pearson correlation analysis was used to assess relationships between BFHHS, implant lateralization, and muscle–tendon lengths. Correlation strength was defined as: |r| = .1-.3 (weak), 0.3-0.5 (moderate), 0.5-0.7 (strong), 0.7-0.9 (very strong), and > 0.9 (excellent/predictive). Univariate linear regression models were used to evaluate associations among BFHHS, implant lateralization, and muscle lengths, with the coefficient of determination (R^2^) reported to quantify model fit.

## Results

### Model performance across diverse pathology

Demographic and anatomical variables are summarized in Table I. Shoulders representing the full spectrum of joint pathology (static subluxation, vault loss, and erosion) were included. The mean adjusted anatomic osteotomy HHS was 47 mm, slightly smaller than the BFHHS (49 mm) derived from geometric fitting (SSM). This difference is consistent with optimization of the modeled sphere, typically resulting in slightly larger BFHHS. Despite this difference, the correlation between the 2 measurements was very strong (r = 0.83, *P* < .0001; power = 0.99), supporting BFHHS as a scalable parameter for surgical planning.

### Best-fit humeral head size predicts lateralization and muscle length

**Figure 3:**
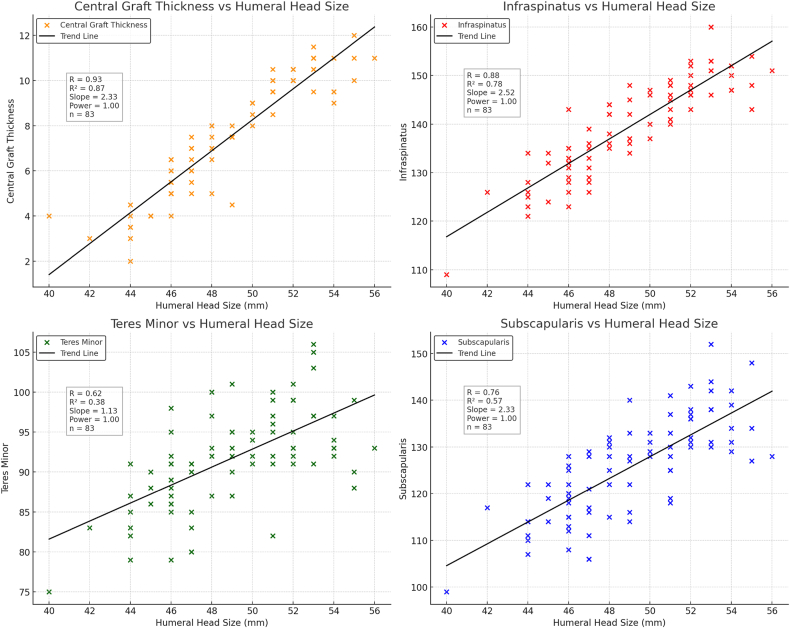
Correlation of humeral head size with baseplate lateralization and rotator cuff muscle length.

BFHHS showed excellent correlation with central graft thickness (r = 0.93, R^2^ = 0.86, *P* < .00001) and posterior cuff muscle lengths: infraspinatus (r = 0.88), teres minor (r = 0.62), and subscapularis (r = 0.76), all *P* < .00001 (Fig. 3). These associations validate the SSM's ability to predict percentage premorbid muscle length even in shoulders with severe wear and subluxation, as previously demonstrated.^23^ The study was powered at 1.0 to detect correlation effect sizes of r ≥ 0.3 (n = 83).

### Patient-specific lateralization strategy based on humeral head size

**Figure 4:**
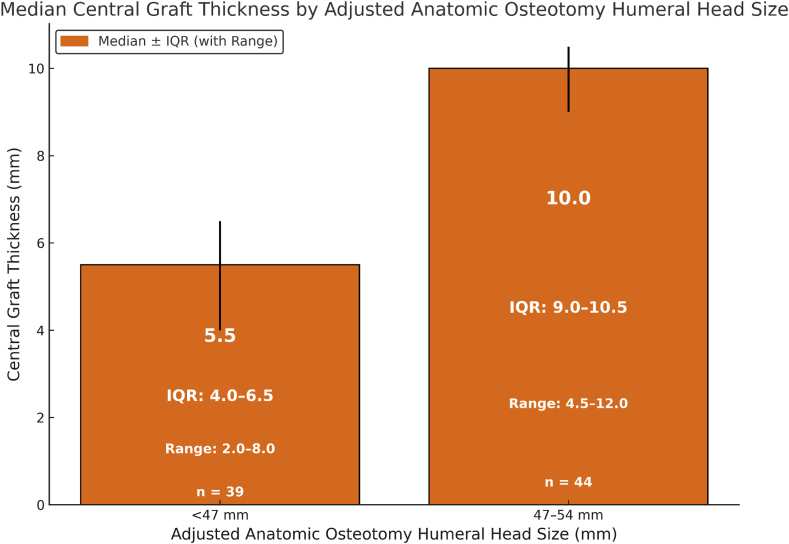
Graft thickness by humeral head size group, showing anatomical basis for lateralization planning. *IQR*, interquartile range.

Patients with smaller humeral heads (adjusted osteotomy HHS <47 mm) had a median central graft thickness of 5.5 mm (interquartile range: 4.0-6.5), while those with larger heads (47-54 mm) required 10 mm (interquartile range: 9.0-10.5) (Fig. 4). This underlines the strong correlation between graft thickness and HHS. The model supports a patient-specific, anatomy-based approach to rTSA lateralization, within limitations imposed by soft-tissue tension.

### Implantation strategy alters muscle–tendon length from pre-operative anatomy

**Figure 5:**
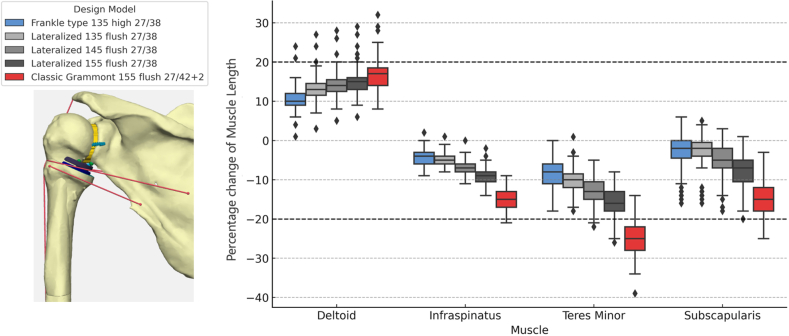
Percent change in muscle length from pre-operative anatomy across implant types. Dashed lines indicate functional muscle length between 80% and 120% according to Blix.^8^ A lateralized Grammont design with a 155° neck-shaft angle is illustrated on the left for visualization purposes.

In the pre-operative anatomic CT scan pose applied to the rTSA configurations, statistically significant differences in percentage muscle–tendon length changes were observed across all 5 implant configurations for each muscle evaluated (Fig. 5; Friedman test: deltoid: χ^2^(4) = 298.59, *P* < .001; infraspinatus: χ^2^(4) = 324.74, *P* < .001; teres minor: χ^2^(4) = 326.80, *P* < .001; subscapularis: χ^2^(4) = 321.20, *P* < .00; Kendall W = 0.9–0.98). Muscle–tendon lengths were expressed as percent deviation from the native anatomic configuration. These findings show that implant geometry altered baseline muscle–tendon length.

The Grammont 155° flush design resulted in the most pronounced shortening of the posterior cuff, particularly infraspinatus and teres minor, and substantial subscapularis shortening, with significant deltoid lengthening. In contrast, the lateralized 135° and 145° flush configurations maintained infraspinatus and subscapularis lengths within ±10% of anatomic muscle length. Teres minor length was maintained within ±10% only with the 135° configurations. The centered Frankle-type 135° preserved deltoid length and achieved a balanced rotator cuff profile. Only the lateralized configurations kept all 4 muscles within ±20% of the anatomic baseline consistent with maintaining muscle–tendon length within physiologic limits.^16^

### Neutral arm position: strategy-dependent differences in muscle–tendon length

**Figure 6:**
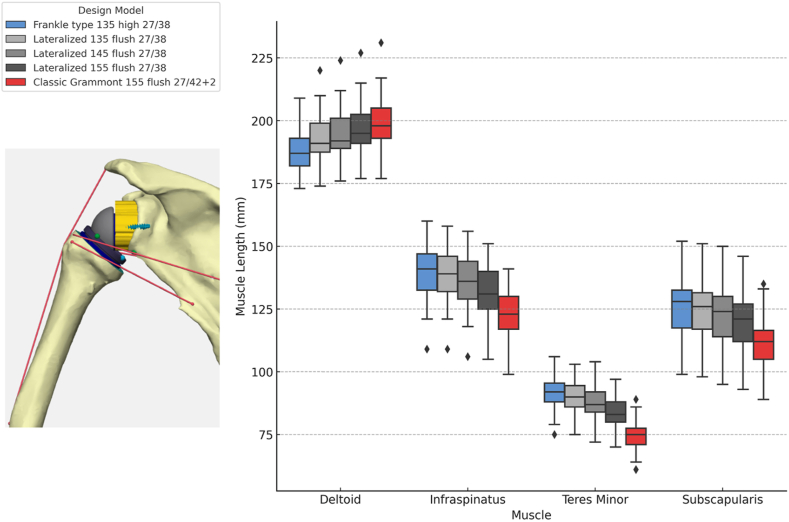
Neutral-arm position, strategy-dependent differences in muscle–tendon length across rTSA configurations. A lateralized Grammont design with a 155° neck-shaft angle is illustrated on the left for visualization purposes.

In the standardized neutral arm position, defined as 0° forward flexion, 0° rotation, and 20° abduction, muscle–tendon lengths differed significantly across implantation strategies for all muscles evaluated.

(Fig. 6; Friedman test: deltoid χ^2^(4) = 294.5, *P* < .001, Kendall W = 0.89; infraspinatus χ^2^(4) = 322.8, *P* < .001, Kendall W = 0.97; teres minor χ^2^(4) = 323.6, *P* < .001, Kendall W = 0.97; subscapularis χ^2^(4) = 317.5, *P* < .001, Kendall W = 0.96). Effect sizes were large for all muscles, indicating robust, strategy-dependent differences in baseline muscle–tendon length.

The centered 135° Frankle-type configuration most closely preserved posterosuperior rotator cuff muscle length relative to the anatomic baseline, particularly for the infraspinatus and teres minor, while maintaining near-anatomic deltoid length. In contrast, the Grammont 155° configuration resulted in pronounced shortening of the posterior cuff muscles, accompanied by relative deltoid lengthening. Lateralized flush configurations with 135° and 145° NSAs demonstrated intermediate muscle–tendon length profiles, preserving posterosuperior cuff length within physiologic limits while maintaining balanced anteroposterior cuff relationships.

## Discussion

This statistical shape and computer model study analyzes a robust cohort of real arthroplasty patients (n = 83) with degenerative pathology, including glenoid erosion, vault loss, subluxation, and cuff deficiency. Using a validated SSM, we quantified rotator cuff and deltoid muscle–tendon lengths in patient-specific CT-derived anatomy across a spectrum of implantation strategies, spanning the evolution from the original Grammont design to more anatomic lateralized configurations. A unique feature of this study is that implant positioning was systematically varied without altering implant geometry, isolating the strategy-dependent geometric effects of the NSA and implantation height while eliminating design confounders.

We tested and confirmed 2 primary hypotheses: (1) that BFHHS predicts the lateralization required to restore near-anatomic muscle–tendon length, and (2) that the SSM reliably estimates muscle length even in the presence of deformity, subluxation, and advanced pathology.

The following key insights emerged:

1. Implantation strategy substantially influences baseline deltoid and rotator cuff muscle–tendon length geometry in silico.^14^
2. BFHHS predicts near-anatomic lateralization requirements and correlates strongly with posterosuperior rotator cuff muscle length, particularly the infraspinatus.
3. The SSM demonstrated robust performance across varied pathology.
4. The Grammont design demonstrates a notable deltoid–rotator cuff length mismatch, a finding consistent with prior biomechanical and clinical observations.^10^^,^^30^
5. More anatomic lateralization strategies better preserve horizontal rotator cuff length relationships.

Implant configuration alone (independent of arm repositioning) significantly influenced muscle–tendon length when compared to premorbid anatomy, underscoring the importance of anatomy-based lateralization strategies for preserving near-anatomic muscle geometry. The centered 135° Frankle-type configuration most closely reproduced near-anatomic overlap-based lateralization and posterosuperior cuff muscle length, preserving infraspinatus, teres minor, and subscapularis excursion within ±10% of the anatomic baseline.^4^^,^^16^ In contrast, the medialized Grammont design resulted in substantial shortening of the horizontal cuff muscles and relative deltoid overlengthening, generating a pronounced mismatch in muscle length. This pattern has been linked to diminished rotational performance in clinical series.^10^^,^^30^ The resulting loss of length of the remaining horizontal rotator cuff places muscle fibers outside the physiological Blix range for optimal force generation,^8^ as previously described by Levin et al.^16^ Lateralized configurations demonstrated intermediate muscle–tendon length profiles, preserving posterosuperior cuff length within physiologic geometric limits while maintaining balanced anteroposterior cuff relationships. These findings emphasize that implantation strategy and lateralization philosophy must be considered together when the objective is preservation of premorbid muscle–length geometry.

While Cabezas et al^11^ confirmed the presence of distinct anatomical size groups and nonuniform scaling relationships among glenohumeral parameters, their findings were not translated into a clinically actionable framework. In contrast, the present analysis builds on these anatomical principles to establish a patient-specific lateralization guide grounded in HHS. BFHHS emerged as a reliable anatomical predictor for the amount of lateralization required to restore near-anatomic muscle–tendon length, enabling direct application to implant planning and decision-making. In our model, BFHHS showed excellent correlation with central graft thickness (r = 0.93) and strong correlations with rotator cuff muscle lengths, including the infraspinatus (r = 0.88), teres minor (r = 0.62), and subscapularis (r = 0.76). These findings align with prior work demonstrating that HHS strongly correlates with baseplate lateralization, supporting a scaled, anatomy-driven approach to reconstruction.^4^

Contemporary implant systems typically offer fixed lateralization steps (eg, 0, +3, or +6 mm), although values vary between manufacturers. Such presets may not adequately reflect the full anatomical spectrum of native shoulder dimensions. The present results suggest that continuous scaling based on HHS better aligns implant configuration with individual muscle–length relationships and may help avoid systematic undercorrection in larger shoulders or overcorrection in smaller shoulders. As a reproducible and intuitive pre-operative metric, CT-derived HHS provides a practical anchor point for anatomy-based rTSA planning.^29^ Although the lateralized inlay models were constructed independently of any specific implant system, the predicted lateralization spectrum aligns closely with the glenoid heads in Frankle's original Altivate design,^23^ supporting the broader generalizability of this framework across systems with comparable anatomical intent.

The SSM demonstrated robust performance across 83 shoulders, including cases with vault loss, static subluxation, and severe deformity. Muscle–tendon trajectories were generated reliably, even in structurally compromised joints, consistent with prior validation by Pitocchi et al^24^ and recent multicenter planning data.^20^ The ability to estimate premorbid muscle length in deformed joints supports the utility of SSM-based pre-operative guidance for individualized rTSA planning.

This study has several limitations. The model is geometric and based on rigid-body assumptions; it does not account for muscle force generation, tendon elasticity, slack length, passive tension, muscle quality such as fatty infiltration or atrophy, or changes in muscle moment arms associated with implant configuration. Accordingly, restoration of near-anatomic muscle–tendon length should be interpreted as an anatomical descriptor rather than a direct surrogate for physiologic tensioning or clinical function. Soft-tissue releases and intraoperative constraints were not modeled and may influence the achievable lateralization in vivo. Finally, while the present framework provides an anatomy-based guide for lateralization planning, clinical validation will be necessary to confirm its utility.

## Conclusion

Implant configuration, including NSA and lateralization strategy, significantly influences muscle–tendon length relationships in reverse shoulder arthroplasty. BFHHS predicts near-anatomic lateralization requirements and posterosuperior rotator cuff muscle length, supporting its use as a patient-specific anatomical reference for implant planning. A centered 135° configuration most closely reproduced the overlap-based anatomic reference, while lateralized–distalized 135° and 145° configurations maintained posterosuperior cuff muscle–tendon length within ±10% of the anatomic reference, corresponding to the physiological Blix length range. These findings establish an anatomy-based, geometry-driven planning framework that warrants future clinical validation.

## Disclaimers:

Funding: No funding was disclosed by the authors.

Conflicts of interest: William G Blakeney is a paid consultant for Enovis/Lima.

Stefan Bauer is a paid consultant for Stryker and Enovis.

Any additional authors, their immediate families, and any research foundations with which they are affiliated have not received any financial payments or other benefits from any commercial entity related to the subject of this article.
